# Functional analysis of the Nep1-like proteins from *Plasmopara viticola*

**DOI:** 10.1080/15592324.2021.2000791

**Published:** 2022-02-12

**Authors:** Jiang Xiang, Jianhui Cheng, Lingzhu Wei, Mingshan Li, Jiang Wu

**Affiliations:** Institute of Horticulture, Zhejiang Academy of Agricultural Sciences, Hangzhou, China

**Keywords:** NLP, *Plasmopara viticola*, cell death, immunity, disease resistance

## Abstract

Necrosis and ethylene-inducing peptide 1 (Nep1) -like proteins (NLP) are secreted by multiple taxonomically unrelated plant pathogens (bacteria, fungi, and oomycete) and are best known for inducing cell death and immune responses in dicotyledonous plants. A group of putative *NLP* genes from obligate biotrophic oomycete *Plasmopara viticola* were predicted by RNA-Seq in our previous study, but their activity has not been established. Therefore, we analyzed the *P. viticola NLP* (*PvNLP*) family and identified seven *PvNLP* genes. They all belong to type 1 *NLP* genes and form a *P. viticola*-specific cluster when compared with other pathogen *NLP* genes. The expression of *PvNLPs* was induced during early infection process and the expression patterns could be categorized into two groups. *Agrobacterium tumefaciens*-mediated transient expression assays revealed that only PvNLP7 was cytotoxic and could induce *Phytophthora capsici* resistance in *Nicotiana benthamiana*. Functional analysis showed that PvNLP4, PvNLP5, PvNLP7, and PvNLP10 significantly improved disease resistance of *Arabidopsis thaliana* to *Hyaloperonospora arabidopsidis*. Moreover, the four genes caused an inhibition of plant growth which is typically associated with enhanced immunity when over-expressed in Arabidopsis. Further research found that PvNLP7 could activate the expression of defense-related genes and its conserved NPP1 domain was critical for cell death- and immunity-inducing activity. This record of *NLP* genes from *P. viticola* showed a functional diversification, laying a foundation for further study on pathogenic mechanism of the devastating pathogen.

## Introduction

Plants have evolved a complicated and multilayered immune system to monitor and cope with infection by diverse phytopathogens. The first layer of plant surveillance system invokes the recognition of evolutionarily conserved molecular signatures derived from various microbes, termed pathogen- or microbe-associated molecular patterns (PAMPs/MAMPs), by employing pattern recognition receptors (PRRs).^1^ A large set of defense responses are subsequently triggered including accumulation of reactive oxygen species (ROS), reinforcement of cell walls, production of antimicrobial substances, activation of mitogen-activated protein kinase (MAPK) cascades, and increased levels of defense-related hormones,^2–4^ collectively resulting in plant resistance to pathogens which is also referred to as PAMP/MAMP-triggered immunity (PTI/MTI). A wide range of microbial patterns or components have been identified and verified as MAMPs from bacteria, fungi, and oomycetes.^5^ However, for virtually MAMPs identified from different kingdoms of life are distinct. For example, flagellin,^6^ EF-TU^7^ and peptidoglycan^8^ are three characterized bacterial MAMPs. Chitin and elicitins act as MAMPs which are secreted from fungi and oomycetes, respectively. Intriguingly, a class of necrosis and ethylene-inducing peptide 1 (Nep1)-like proteins (NLPs) have been described as MAMPs from mostly phytopathogens across all three of the taxonomically unrelated groups.^9–11^

The founding member of NLPs, a 24-kDa protein, was isolated in 1995 from *Fusarium oxysporum* culture filtrates based on its capability to induce necrosis and ethylene biosynthesis in numerous dicotyledonous but not in monocotyledonous plants, therefore named after necrosis and ethylene inducing peptide 1 (Nep1).^12^ Since then, an emerging group of Nep and Nep1-like proteins (NLPs) were identified from various phytopathogenic microorganisms including bacteria, fungi, and oomycete by the presence of a common NPP1 (necrosis-inducing *Phytophthora* protein 1) domain.^13–17^ A hallmark of conserved NPP1 domain is a heptapeptide motif “GHRHDWE” in the central region of the NLPs and two to six cysteine residues. Base on the number of cysteine residues, NLPs can be classified into types 1, 2, and 3, which possess two, four, and six cysteine residues, respectively.^9,18^ In addition, type 1 NLPs contain a noncytotoxic subgroup of NLPs (designated type 1a) which can be distinguished according to the amino acid substitution in the cation-binding pocket.^18^ The number of *NLP* gene family members can vary significantly among diverse taxonomy of microorganisms. For instance, the Arabidopsis pathogen *Hyaloperonospora arabidopsidis* have 12 *NLP* genes, and soybean pathogen *Phytophthora sojae* have a total number of 33 *NLP* members, whereas only a single *NLP* gene has been identified from the genome of wheat pathogen *Mycosphaerella graminicola*.^19–21^ Comparative genome analysis among the already fully sequenced genomes of a broad range of oomycetes and fungi revealed that the *NLP* family in oomycetes is more expanded than in fungi pathogens.^19^

It is well known that NLPs are capable of stimulating plant immunity-associated defenses in dicotyledonous but not in monocotyledonous plants.^22–24^ Thus, NLPs were proposed to have dual function in plant-pathogen interaction, i.e., activating immune responses and acting as a toxin-like virulence factor which inducing plant necrosis.^25^ Cytotoxic NLPs were found to trigger defense-related genes expression in *Arabidopsis thaliana*, which clearly overlaps with that activated by flg22,^15,23^ suggesting the similarity of cytotoxic NLP-triggered immune response to the MAMP-triggered immunity (MTI). Furthermore, cytotoxic NLPs also induce a myriad of other defense responses in plants including activation of mitogen-activated protein (MAP) kinase activity, deposition of callose and increased production of reactive oxygen species.^20,23,26^ However, not all NLPs have the ability to induce necrosis which are called noncytotoxic proteins. When tested by transient expression in tobacco, necrosis was only induced by 2 out of 9 NLPs from *Verticillium dahlia*,^27^ 8 out of the 30 NLPs of *Phytophthora sojae*,^21^ whereas none of the 10 tested NLPs of *H. arabidopsidis* were able to cause necrosis, including the host *A. thaliana*.^19^ These observations indicate that NLPs may have roles independent of cytotoxicity. For the hemibiotrophic pathogens *P. sojae and P. infestans*, cytotoxic *NLPs* were expressed during the infection stages coinciding with the transition from biotrophy to necrotrophy.^28,29^ In contrast to cytotoxic *NLPs* which are mainly detected at the necrotrophic stages of infection, most noncytotoxic *NLPs* appear to be expressed during early stages of infection,^19,28^ suggesting they may function in penetration or initial colonization of the host. Several observations showed that NLPs from different pathogens play distinct roles in pathogen virulence. In some cases, NLPs were regarded as positive virulence factors that facilitated pathogen growth in hosts through disintegration of host´s membrane, resulting in serious plant diseases. The virulence of *Colletotrichum coccodes* was increased by transformed with *NEP1* gene from *Fusarium oxysporum* and the spectrum of hosts also be infected.^30^ Also, transformation of the *NLP*-deficient mutants of *Pectobacterium carotovoum* with *NLP_Pya_* or *NLP_Pp_* showed 30%-40% restoration in virulence and maceration of potato tuber.^31^ The deletion of NLP in *Erwinia carotovora* attenuated virulence on both rubbers and stems of potato.^32^ However, NLP-deficient mutants of *Botrytis elliptica, Mycosphaerella graminicola, Verticillium dahliae*, and *Magnaporthe oryzae* were not reduced in virulence on each host, indicating *NLP* genes are not virulence determinants in these plant pathogen interactions.^20,24,26,27^

In the current publication we provide an analysis of the potential biological roles of the 7 *PvNLP* genes that have been previously identified in *Plasmopara viticola*, the causal agent of grapevine downy mildew, whose transcriptome- and genome-sequencing were completed in our previous work.^33,34^ The objectives of this study were to define variation in their function, to use agroinfiltration assays to determine whether any of them play important roles in cell death-inducing activity or immune responses, and to generated *PvNLP*-expressing Arabidopsis plants to determine whether any of them have the ability to trigger immunity as a MAMP.

## Materials and methods

### Plant material, strains, and growth condition

The grapevine (*V. vinifera* cv. Thompson seedless) and *Nicotiana benthamiana* used in this study was grown in a greenhouse at 25°C with 16 h of illumination per day. The *Arabidopsis thaliana* (Col-0) were cultivated in a phytochamber with a photoperiod of 22°C/16 h light and 16°C/8 h darkness under 40%-60% humidity. Highly virulent *Plasmopara viticola* strain ZJ-1-1 has been tested in our previous study and routinely subcultured on grapevine leaf discs every 10 days at 22°C/16 h light and 16°C/8 h dark cycles. *Agrobacterium tumefaciens* strain GV3101 carrying the disarmed Ti plasmid was cultured on Luria-Bertani (LB) agar or broth medium at 28°C.

### RNA isolation, cDNA synthesis and quantitative RT-PCR

To monitor *PvNLPs* transcript profiling during *P. viticola* infection of grapevine, leaf inoculation using spore drops of *P. viticola* ZJ-1-1 and total RNA was extracted using the CTAB procedure were conducted as previous described.^35^ For RNA isolation of *N. benthamiana*, a commercial kit (RNA simple Total RNA Kit, Tiangen) was used following the recommended protocols. All cDNA synthesis and quantitative RT-PCR reaction were performed by using protocols established in our lab. Briefly, the total RNA was used as a template for reverse transcription with a RevertAid TM First Strand cDNA Synthesis Kit (Invitrogen, Carlsbad, CA, USA). The qRT-PCR was performed on the QIAGEN Rotor-Gene Q system (QIAGEN, Hilden, Germany) using a SuperReal PreMix Plus (SYBR Green) kit (TIANGEN Biotech Co., Ltd., Beijing, China). Primers (Table S1) were designed to anneal specifically to each targeted gene and by using Beacon Designer 8.14 software with default setting for SYBR Green real-time PCR. The *PvActin* gene^36^ and *NbEF1α* gene^35^ were used as constitutively expressed endogenous controls to determine relative expression values by the 2^−ΔΔCt^ method^37^ for *P. viticola* and *N. benthamiana*, respectively.

### Gene cloning and sequence analysis

The coding sequences of the PvNLPs were amplified using cDNA from *P. viticola* isolate ZJ-1-1 based on our previous RNA-seq results,^33^ cloned into a pLB vector ((Tiangen, Beijing) and verified by PCR and Sanger sequencing. The gene-specific primers for PvNLPs cloning are listed in the Table S1. The potential signal peptides of PvNLPs were predicted with online software SignalP 5.0 (http://www.cbs.dtu.dk/services/SignalP/). Multiple sequence alignment of NLPs from three different kingdoms of life was carried out using software MEGA 7.0 (Oxford University, England, UK). The phylogenetic relationship analysis of three different types of NLPs was done using the Neighbor-Joining method,^38^ with 1000 bootstrap replicates.

### Construction of expression plasmids

The open reading frame sequences of seven genes were amplified from the pLB-PvNLPs with primers containing restriction enzyme cutting site (Table S1). To make the stable expression recombinant vectors pHB-PvNLPs-flag, the PCR fragments were digested with *Bgl II*/ *Bcl I* and *Spe I* restriction enzymes which are isocaudarners of *BamH I* and *Xba I*, respectively. The binary vectors pHB-flag was cut using *BamH I* and *Xba I* restriction enzymes. Then the digestion fragments of PvNLPs were ligated into the digested pHB-flag vectors. To generate the pGR106-PvNLPs-flag for PVX and Western blot assays, the fragments harboring flag tag were amplified from pHB-PvNLPs-flag and cut with *Asc I* and *Not I* restriction enzymes and inserted into the pGR106 vector. All the generated plasmids were verified by Sanger sequencing by Majorbio, Inc. (Shanghai, China).

### Agrobacterium*-mediated transient expression in* Nicotiana benthamiana and *pathogen infection assays*

Constructs were transformed into *A. tumefaciens* strain GV3101 by electroporation and cultured in LB medium supplemented with the appropriate antibiotics (12.5 μg/ml tetracycline, 50 μg/ml kanamycin and 50 μg/ml rifampicin) at 28°C for 2 days.^39^ For cytotoxicity assay, *Agrobacteria* harboring constructs of pGR106-PvNLPs were cultured in LB broth medium containing 50 μg/mL of kanamycin and 12.5 μg/ml of tetracycline at 28°C with shaking at 200 rpm for 60 h. The culture was harvested by centrifugation and washed three times using 10 mM MgCl_2_, then resuspended in 10 mM MgCl_2_ to achieve a final OD_600_ of 0.4. After incubation for 1 h at 28°C, then 1 mL of *A. tumefaciens* cell suspension was infiltrated into plant leaves by using needleless syringes. Symptom development was monitored 4–8 dpi in *N. benthamiana*. Each assay consisted of at least three plants (40 days old) inoculated on four leaves. For *Phytophthora capsici* infection, zoospores were prepared as described and the infection assays were conducted as our previous study.^40,41^ Lesion diameters were determined by manually measuring with vernier caliper.

### Electrolyte leakage assays

Cell death of *N. benthamiana* leaves was quantitatively assessed by measuring ion leakage of leaf discs as previously described.^42^ In brief, for each sample, three leaf discs (8 mm diameter) cut from inoculated leaves using a cork borer were placed into a 50-ml tube containing 5 ml sterile and double-distilled water for 3 h at room temperature (RT). Then the conductivity EC1 of the bathing solution was measured using the sensor of an electrical conductivity meter (DDS-307, Rex Shanghai, China). The tubes containing the solution and leaf disks were then boiled for 25 min. After cooling to room temperature, the conductivity EC2 of the solution was measured. Electrolyte leakage (%) = 100 × EC1/EC2. These experiments were repeated for a total of three times.

### Western blot assays

Agroinfiltrated *N. benthamiana* leaves were harvested at 2 dpi and homogenized in liquid nitrogen. And then 1 mL lysis buffer was added to 600 mg of each ground sample. The samples were mixed gently and centrifuged at 12500 rpm for 10 min at 4°C. Supernatants were obtained and separated by 12% sodium dodecyl sulfate-polyacrylamide gel electrophoresis (SDS-PAGE) and transferred to a nitrocellulose blotting membrane. Western blot analysis was conducted using an anti-cFlag peroxidase conjugate (Sigma-Aldrich).

#### A. thaliana *stable transformation and pathogenicity assays*

*Agrobacterium tumefaciens* carrying the empty vector pHB-flag or pHB-PvNLP-flag was cultured, harvested and resuspended in a solution of 5% sucrose and 0.02% Silwet L-77 (Lablead Biotech Co., Ltd., Beijing, China). Arabidopsis wild-type plants (Col-0) were transformed by dipping flowers into the suspension as described previously.^43^ The hygromycin-resistant seedlings were screened on selective medium containing 30 μg/mL of hygromycin (Sigma-Aldrich) for 2 weeks and transplanted in soil. Positive transgenic plants were validated by semi-quantitative PCR.

Infection assays on Arabidopsis seedlings were performed with downy mildew pathogen *Hyaloperonospora arabidopsidis* (50 spores per μL). To collect spores for inoculation, wild-type (Col-0) leaves with sporulating *Hyaloperonospora arabidopsidis* were harvested 7 dpi and washed with deionized H_2_O by vortexing in tubes. The spore suspension was then filtered and sprayed onto the 14-days-old plants of transgenic and wild-type Arabidopsis using a mini spraying device. Subsequently, plants were left to dry for about 30 minutes and cultured at 100% humidity under long day conditions (16 h light, 18°C). The disease severity or zoospores growth was quantified 6 dpi. For zoospores counting, the aerial parts of seedlings were cut and suspended in a known volume of water and the total number of zoospores per milligram of plant tissue was measured.

## Results

### *Identification of the NLP family in* P. viticola

**Figure 1.**
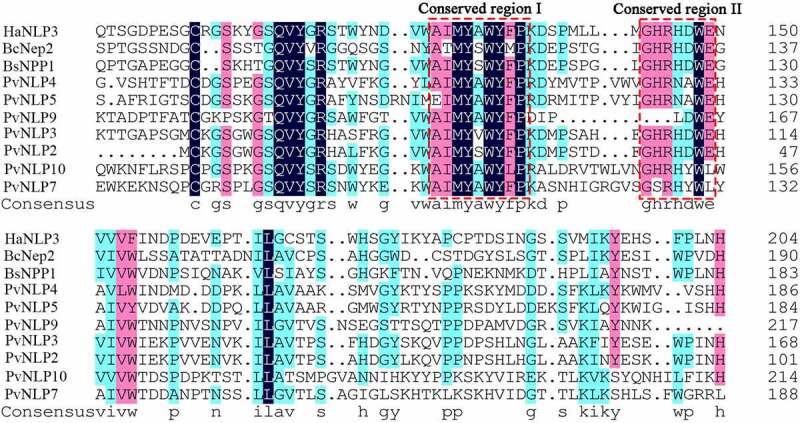
Multiple sequence alignment of PvNLPs with other pathogens NLP proteins.

In our previous work, the secretomes of three different *P. viticola* isolates (JL-7-2, ZJ-1-1 and CSIRO-L-2) were explored by de novo transcriptome analysis.^33^ A total number of 17 NLP coding genes were predicted in these three *P. viticola* isolates. After removing duplicate genes and incorrect genes which have no typical conserved motifs of NLP proteins, there are 7 *PvNLP* putative genes left. Then the full-length of *PvNLP*s were cloned from the cDNA of pathogen isolate ZJ-1-1 for further functional evaluation. None of them has an intron which was confirmed by cloning genes from genome of *P. viticola* isolate ZJ-1-1. The protein sequences of these PvNLPs, ranging in size from 223 to 279 amino acids, were submitted to SignalP 5.0 for secreted signal peptide prediction. PvNLP4, PvNLP5, PvNLP7, PvNLP9 and PvNLP10 had a signal peptide consisting of 17 to 21 amino acid residues (Table S2), which was predicted to regulate the secreted proteins. Whereas PvNLP2 and PvNLP3 did not have a predicted signal peptide and therefore may not be secreted into the apoplast in native mycelia. In our research, PvNLP1, PvNLP6, and PvNLP8 were missing compared with the previous study.^44^ It is possible that the expression of these three genes was not detected in our transcriptome or the transcriptome sequencing is not deep enough. On the basis of multiple sequence alignment of PvNLPs with other pathogen NLPs revealed they shared the conserved 24-aa peptide (nlp24) containing conserved region (AIMYAWYFPKD) and (GHRHDWE) at the C-terminal end (Figure 1). These characteristics were demonstrated to be sufficient to trigger immune response in plants and can identify any new peptide sequence as an NLP in pathogens^10−11^.

In the central region, the conserved region I and conserved region II are indicated by the red boxes. The similar amino acids are shaded (100%: black, 75%: purple; 50%: light blue).

**Figure 2.**
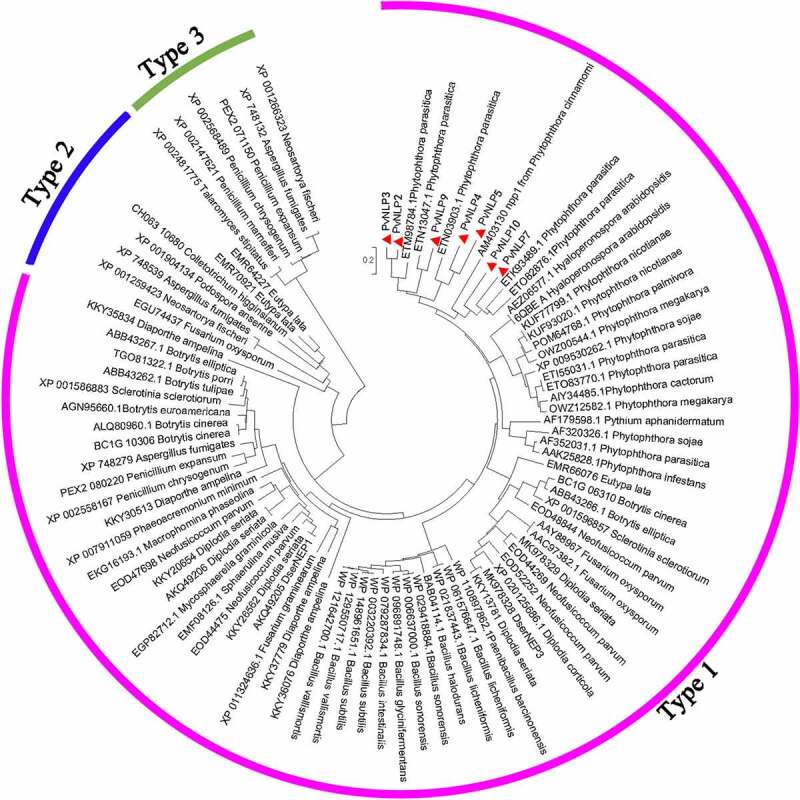
Phylogenetic tree analysis of PvNLPs with other plant pathogen-derived necrosis- and ethylene-inducing protein (Nep1)-like proteins (NLP).

To determine the relationship of PvNLPs to the reported NLP subfamilies, a maximum likelihood phylogenetic analysis of PvNLPs was conducted together with representatives of type 1, type 2 and type 3 NLP proteins in various pathogens including fungi, oomycetes and bacteria. Phylogenetic analysis showed that PvNLPs were all grouped together with type 1 NLPs whereas type 2 and type 3 NLPs were separated into different groups (Figure 2). These results indicating the seven PvNLPs most probably belong to type 1 NLP proteins which were found almost exclusively in plant-associated microbes, whereas type 2 and type 3 NLPs were found in microorganisms with different lifestyles.^10^

The phylogenetic tree was constructed based on neighbor-joining method using MEGA 7.0 with 1000 bootstrap replicates. The scale bar indicates 0.2 substitutions per site. Genbank accession numbers of NLP proteins from different species are shown in the figure and the accession numbers of PvNLPs are listed in Table S3.

### *Expression profiles of* PvNLP *genes*

**Figure 3.**
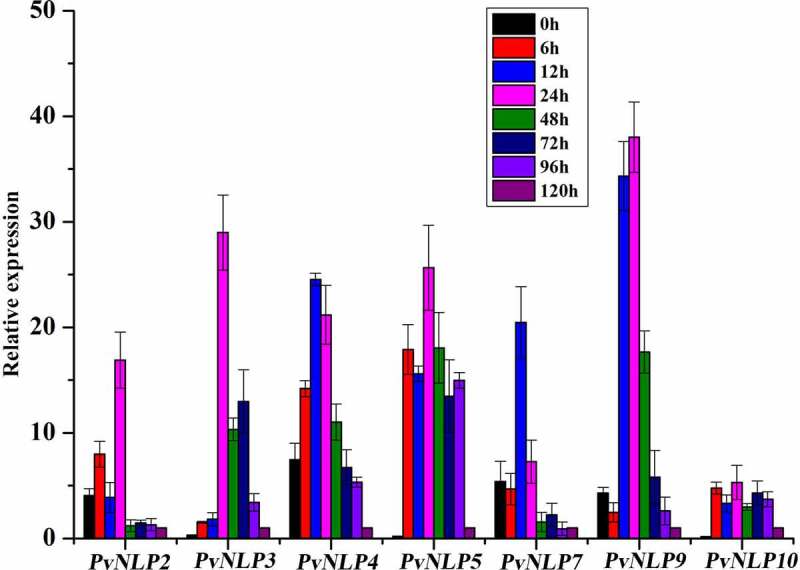
Transcriptional expression of 7 *PvNLP* genes from *P. viticola* isolate “ZJ-1-1” during the pathogen-grapevine infection.

To determine the potential role of PvNLPs during the interaction between *P. viticola* and its host plants, we inoculated susceptible grapevine leaves with strain ZJ-1-1, and analyzed transcriptional patterns for *PvNLPs* at different time points (0, 6, 12, 24, 48, 72, 96 and 120 hpi) after infection by quantitative real-time PCR. Results showed that all seven members of the *PvNLP* family were strikingly up-regulated during the early stages of infection process but with seemingly distinct activation patterns. Four genes (*PvNLP2, PvNLP4, PvNLP7*, and *PvNLP9*) were already highly expressed at 0 hpi before the interaction with the grapevine leaves. *PvNLP5* and *PvNLP10* were strongly induced at 6 hpi and reached a maximum level at 24 hpi. Whereas a significant transient induction of PvNLP3 were detected at a relatively late time point (24 hpi) (Figure 3). According to their temporal expression patterns during infection, the *PvNLPs* genes could be grouped into two kinds. In general, the first group, comprising *PvNLP2, PvNLP3, PvNLP7* and *PvNLP9*, was expressed strongest at the early stages of infection (up to 24 hpi) but subsided quickly afterward. However, the second group (*PvNLP4, PvNLP5* and *PvNLP10*) genes were highly expressed at multiple time points in both early and late stages almost the entire infection process. Although previous study showed that *PvNLP2* and *PvNLP3* expression also increased during the first 6 h of the infection, the expression stayed elevated during the whole infection and both reached a maximum level at 72 hpi^44^ which was different from our results. It may be resulted from the different isolates of *P. viticola* and grapevine varieties.

Relative *PvNLPs* mRNA levels were quantified by quantitative RT-PCR in samples and grapevine leaves inoculated with *P. viticola* at different time-points post infiltration. *P. viticola actin* transcripts were used as a reference. Error bars represent standard deviation of three technical replicates. Similar results were obtained in three independent experiments.

### *Cytotoxicity of PvNLPs in* N. benthamiana *leaves*

**Figure 4.**
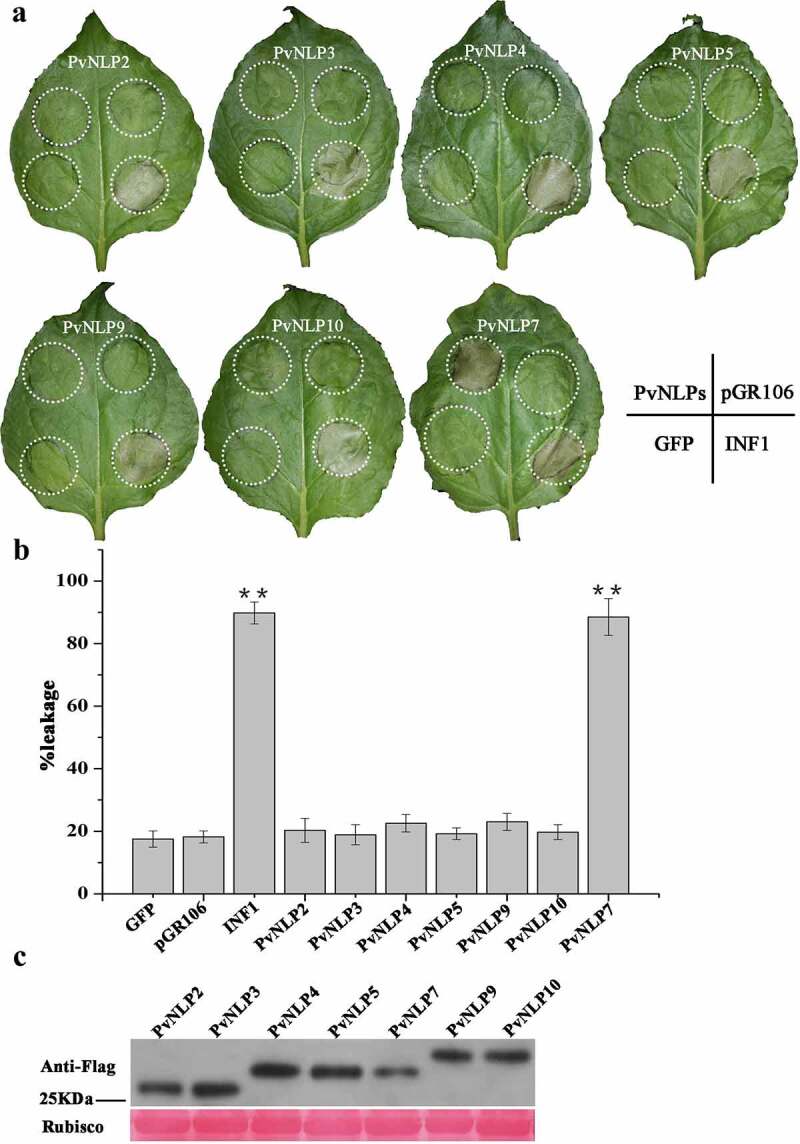
Comparative analysis of necrosis-inducing response observed for various *PvNLP* genes by PVX agroinfiltration in *N. benthamiana* leaves.

Several NLPs from various microorganisms have been shown to be able to induce necrosis cell death preferentially in dicotyledonous plants. Nevertheless, the ability to induce necrosis of NLPs was not always consistent in many pathogens, such as the obligate biotroph *Hyaloperonospora arabidopsidis*. In order to investigate the PvNLP-induced cellular responses in *N. benthamiana* plants, we transiently expressed the seven different PvNLP coding sequences by using agroinfiltration, a system that has been previous validated to determine cytotoxic activity of NLP proteins.^45^ INF1 from *P. infestans*, which is known to induce cell death in *N. benthamiana*, was served as positive control^46^ and GFP^35^ and empty vector pGR106 were negative controls. Figure 4a showed that only PvNLP7 was able to induce necrosis in *N. benthamiana* leaves 8 days after infiltration, showing the similar symptom to that induced by INF1. The other six PvNLP proteins did not result in any cell death response as did the negative controls GFP and pGR106 (Figure 4a and b), with no visible symptoms up to 2 weeks post-inoculation. Immunoblot analysis of PvNLPs showed that all *PvNLP* genes were expressed normally in *N. benthamiana* (Figure 4c). These results indicated that PvNLPs differed in cytotoxicity in *N. benthamiana*.

**(A)** Leaves were infiltrated with *A. tumefaciens* harboring the pGR106 vector containing various *PvNLP* genes and the indicated controls. Each leaf was co-inoculated with INF1 as a positive control. The GFP and empty vector pGR106 were used as negative controls on the same leaf. Circles indicate the disservice regions around the point of infiltration. The photography was taken 8 d post infiltration. The experiment was repeated three times with similar results. **(B)** Quantification of cell death by measuring electrolyte leakage. Error bars represent standard errors from three biological replicates (**, *P* < .01, Dunnett’s test). **(C)** Immunoblot analysis of proteins from *N. benthamiana leaves* transiently expressing *PvNLPs*.

### *PvNLP induces disease resistance in* N. benthamiana

**Figure 5.**
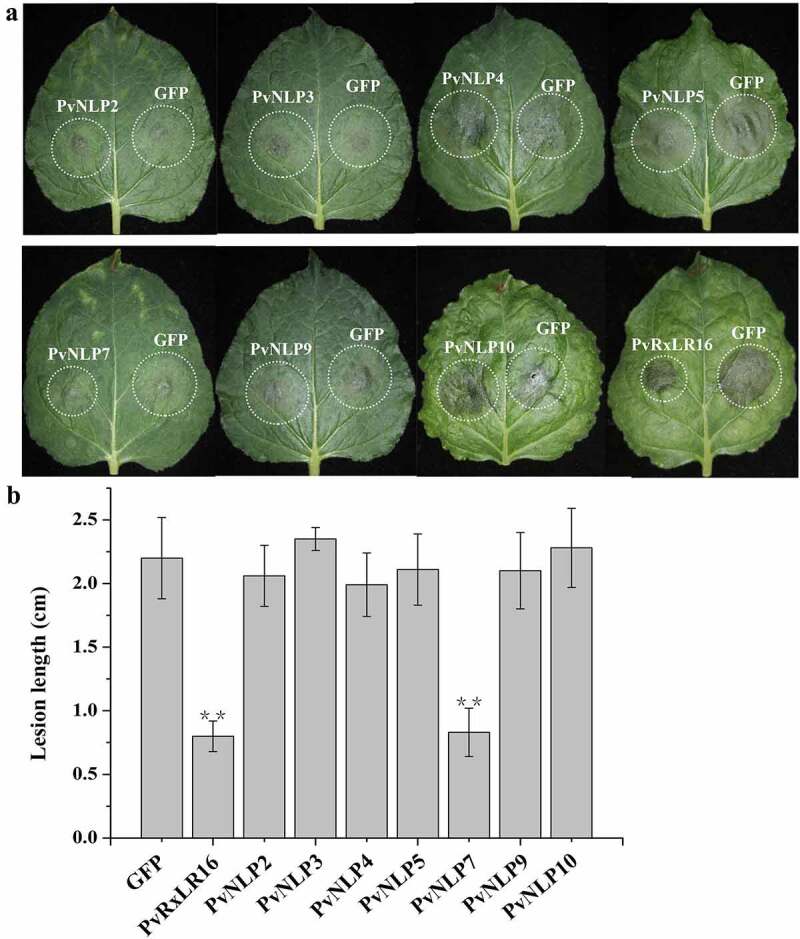
Disease resistance induction in *N. benthamiana* by PvNLPs.

NLP proteins, secreted from multiple pathogens, were proposed to have the function of triggering many characteristic immune responses in dicotyledonous plants.^27,47,48^ To determine whether PvNLPs could trigger defense responses in plants, the leaves of *N. benthamiana* were agroinfiltrated with separately PvNLP, and GFP was used as a control. The *Agrobacterium* suspension was adjusted to a non-lethal concentration (OD_600_ = 0.2) which could not induce cell death in *N. benthamiana* at 3 d post-infiltration. The infiltrated regions were inoculated with *P. capsici* zoospores after 24 h of agroinfiltration. The disease resistance induced by transient *PvNLPs* expression was evaluated by calculating lesion diameter after 36 h of inoculation. The lesion diameter was significantly smaller in *PvNLP7* than *GFP*-expressing leaves, whereas the remaining 6 *PvNLP-*expressing leaves showed similar levels of disease symptom with control (Figure 5a and b). Therefore, *PvNLP7* could induce *P. capsici* resistance in transiently transformed *N. benthamiana*.

**(a)** Lesions of the *N. benthamiana* leaves expressing the indicated genes inoculated with *P. capsici* at 36 hpi. The PvRxLR16 and GFP were used as a positive and a negative control, respectively. The experiment was repeated three times with similar results. **(b)** Lesion diameters of *N. benthamiana* leaves (**P < .01, Dunnett’s test).

### PvNLPs *expression in arabidopsis leads to severe growth reduction and enhances plant resistance to downy mildew*

**Figure 6.**
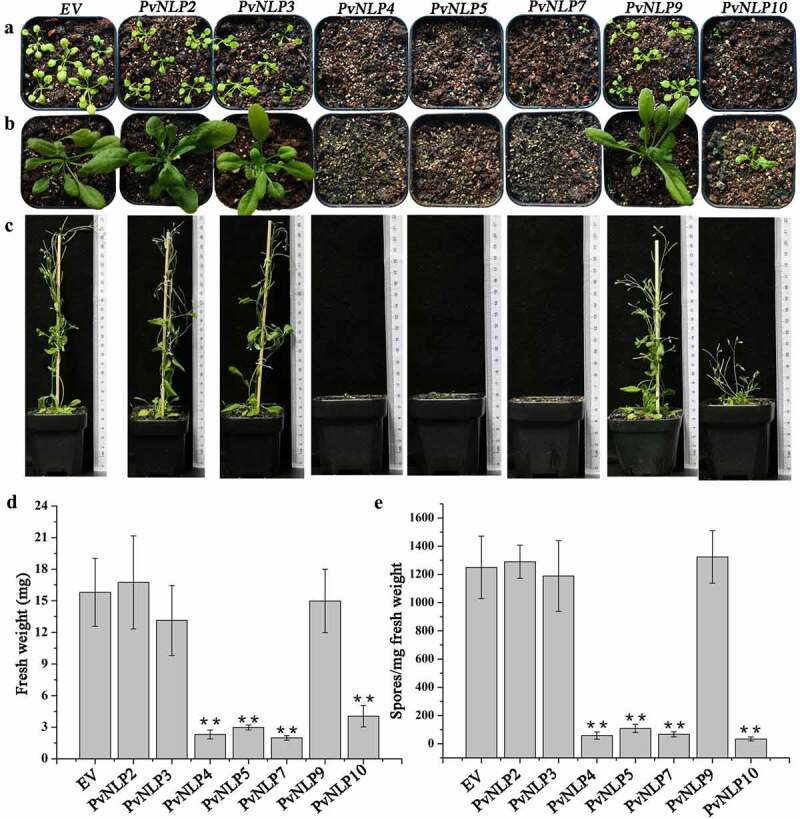
*PvNLPs* expression in Arabidopsis leads to growth reduction and enhanced resistance to downy mildew.

The aforementioned experiments demonstrated PvNLP proteins have the abilities to improve *N. benthamiana* disease resistance. To verify whether PvNLPs enhance disease resistance in other dicotyledonous plants as in *N. benthamiana*, they were fused with the N terminus of flag under the control of the CaMV 35S promoter and stably expressed in Arabidopsis. All seven *PvNLP* genes transgenic lines were generated by hygromycin resistance selection and verified by DNA PCR and qRT-PCR detection. Surprisingly, ectopic expression of 4 of the 7 *PvNLP* genes (*PvNLP4, PvNLP5, PvNLP7*, and *PvNLP10*) resulted in transgenic Arabidopsis showing aberrant phenotypes such as dwarfed and severely reduced growth, compared with control plants transformed with empty vector (Figure 6a, b and c). Some severely abnormal transgenic plants even stopped growing with only two purple cotyledons, but some weakly aberrant plants did transition to flowering and produced seeds for homozygous screening (T3). However, the remain *PvNLPs* (*PvNLP2, PvNLP3*, and *PvNLP9*) did not show any significant differentiation in phenotypes in contrast with flag-expressing control. All *PvNLPs-*expressing lines growth was evaluated in the T3 generation by determining weight of the aerial parts of 20 seedlings per transgenic line. The *PvNLP4-, PvNLP5-, PvNLP7-*, and *PvNLP10-* expressing lines showed significant weight reduction compared with other *PvNLPs* and control plants, which confirmed the growth effects observed on individual T3 generation plants (Figure 6d).

**(a)** Growth phenotype of representative T3 transgenic Arabidopsis lines (14 d old) expressing *PvNLP* genes. **(b)** Growth phenotype of representative T3 transgenic Arabidopsis lines (28 d old) expressing *PvNLP* genes. **(c)** Growth phenotype of representative T3 transgenic Arabidopsis lines (45 d old) expressing *PvNLP* genes. **(d)** The reduction in growth was quantified as fresh weight of the aerial parts of T3 seedlings. **(e)** Sporulation level of *H. arabidopsidis* on *PvNLPs* transgenic lines. Arabidopsis seedlings were spray-inoculated with *H. arabidopsidis* at 14 d after germination. Seedlings were harvested and spores were measured at 6 d post inoculation (6 dpi). Data are shown as the mean of biological triplicates ± SD (n = 6). Asterisks indicate significant difference (**, *P* < .01, Dunnett’s test).

To investigate the role of *PvNLPs* in the process of resistance to the downy mildew *H. arabidopsidis*, we inoculated living plant seedlings with the pathogen and counted spore number per milligram of different transgenic lines. *PvNLPs*-expressing Arabidopsis plants showed enhanced resistance to the pathogen, and strikingly, these same transgenic lines also showed severe growth inhibition (Figure 6e). It seems that there was a strong correlation between the level of resistance and the fresh weight of different *PvNLPs*-expressing lines. In previous reports, there is a great many instances of plant growth suppression as a result of triggering plant immune response.^18,49^ The fact that the level of immunity of the transgenic lines expressing *PvNLP* genes is strongly correlated to their growth reduction, therefore, indicates that PvNLPs triggered immunity in Arabidopsis plants resulting in the observed growth reduction.

### The NPP1 domain of PvNLP7 is essential for inducing cell death and disease resistance

**Figure 7.**
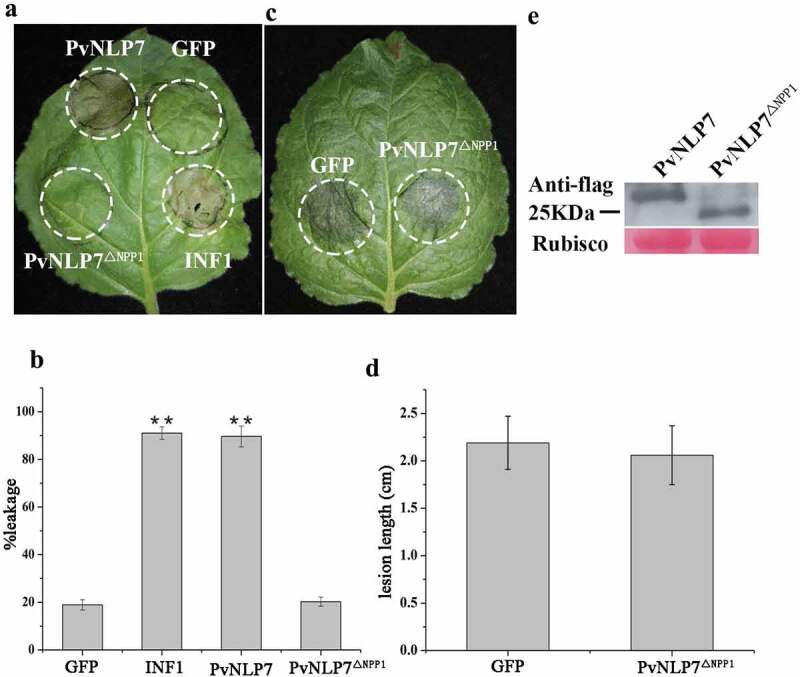
The NPP1 domain (a 27-aa peptide) of PvNLP7 is essential for inducing necrosis and disease resistance.

It is well known that NLPs share a conserved domain referred as the NPP1 domain (also called nlp20 peptide) in diverse microbial pathogens.^14^ In an attempt to investigate whether the NPP1 domain of PvNLP7, containing conserved region I and region II (shown in Figure 1), has effect on the cell death-inducing or immunity activity, deletion mutant PvNLP7^ΔNPP1^ lacking NPP1 domain was tested in *N. benthamiana*. As expected, PvNLP7^ΔNPP1^ could not induce cell death as wild type anymore (Figure 7a and b). Likewise, the ability of enhancing disease resistance to *P. capsici* was abolished (Figure 7c and d). The expression of PvNLP7 and truncated protein PvNLP^ΔNPP1^ were checked by Western blot (Figure 7e). This finding highlights the importance of this NPP1 domain for the cell death-activation and disease resistance activity of PvNLP7.

**(a)** Comparative analysis of necrosis-inducing response observed for PvNLP7 and deletion mutant PvNLP7Δ^NPP1^ by PVX agroinfiltration in *N. benthamiana* leaves. The INF1 and GFP were used as a positive and a negative control on the same leaf, respectively. Circles indicate the disservice regions around the point of infiltration. The photography was taken 8 d post infiltration. The experiment was repeated three times with similar results.**(b)** Quantification of cell death by measuring electrolyte leakage. Error bars represent standard errors from three biological replicates (**, *P* < .01, Dunnett’s test).**(c)** Comparative analysis of disease resistance induction in *N. benthamiana* by PvNLP7 and deletion mutant PvNLP7Δ.^NPP1^ Lesions of the *N. benthamiana* leaves expressing the indicated genes inoculated with *P. capsici* at 36 hpi. The GFP was used as a negative control. The experiment was repeated three times with similar results.**(d)** Lesion diameters of *N. benthamiana* leaves (**P < .01, Dunnett’s test).**(e)** Immunoblot analysis of proteins from *N. benthamiana* leaves transiently expressing PvNLP7 and deletion mutant PvNLP7Δ.^NPP1^

### PvNLP7 enhances the expression of defense-associated genes

**Figure 8.**
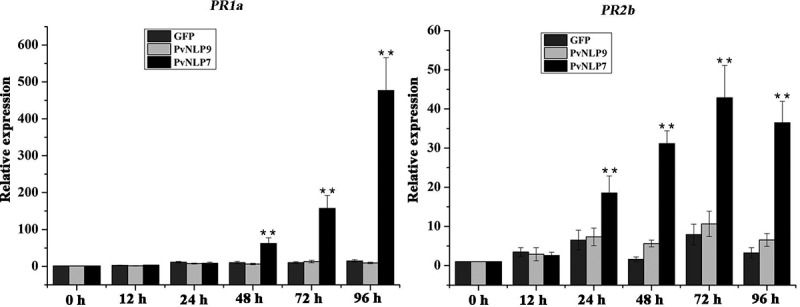
Upregulation of defense related genes mediated by PvNLP7 in *N. benthamiana.*

To increase our understanding of the role of PvNLPs in plant disease resistance, PvNLP7, the only one inducing cell death in *N. benthamiana* was chosen for further study. GFP and PvNLP9 which could not trigger immunity in *N. benthamiana* and Arabidopsis were selected as negative controls. The transcriptional levels of two defense-associated genes, *PR1b* and *PR2b*, were monitored in *N. benthamiana* transiently expressing *PvNLP7* using real-time qRT-PCR. The *PR1b* and *PR2b* are both marker genes for salicylate-mediated signaling which play a vital role in defense resistance to pathogens.^50,51^ The relative expression levels of *PR1b* and *PR2b* induced by PvNLP7 began to increase significantly compared to controls at 48 h and 24 h, and then reached peaks at 96 h and 72 h, respectively (Figure 8). Whereas the PvNLP9 and GFP slightly or even not induced the expression levels of *PR1b* and *PR2b*. These results revealed that PvNLP7 recognition by plants may induce the expression of defense-associated genes, resulting in disease resistance.

Transcript level of the *PR1a* and *PR2b* genes induced by PvNLP7 at different time points. The Agrobacterium suspension harboring PvNLP7 was adjusted to a non-lethal concentration (OD_600_ = 0.2) before infiltration. Means and standard errors from three independent replicates are shown (**P < .01, Dunnett’s test).

## Discussion

In this report, we described the characterization of seven independent *NLPs* genes from the obligate biotrophic pathogen *P. viticola*. They were all cloned and classified as type 1 NLPs, like all other oomycete NLPs characterized so far.^9,18^ Five members of the *NLP* gene family in *P. viticola* have been identified elsewhere,^44^ but only two (*PvNLP2* and *PvNLP3*) were selected for functional analysis. Considering these new findings, the *PvNLP* family comprises a total of 10 genes and is more expanded in *P. viticola* than initially described.

It is well known that NLPs typically possess a signal peptide followed by a nlp24 peptide. *PvNLP* genes encode predicted secreted proteins that are likely targeted to the host apoplast during infection. Intriguingly, *PvNLP2* and *PvNLP3* were predicted to have no signal peptide (SP) and SP cleavage site based on SignalP HMM probability test. It is possible that the algorithms of online software used for prediction of SP are not accumulate or accurate for oomycete sequences. The secretory function of these two genes needs to be further confirmed by signal sequence trap assay (SST) in the future. The nlp24 peptide is strongly conserved in the type 1 of both cytotoxic and noncytotoxic NLPs, which can trigger plant immune responses^10−11^. All studied NLPs from *P. viticola* contain the nlp24 peptide with variable degeneracy, which of PvNLP9 has only a minimum of 16 amino acids (Figure 1). Previous studies showed that the peptide is indispensable for necrosis-inducing activity^10−11^. We discovered that the nlp24 peptide of PvNLP7 is essential for phytotoxic activity and immune responses, which strengthen the importance of nlp24 peptide in the interaction between microorganisms and plants.

*NLP* genes from different pathogens expressed under certain conditions have been reported in several cases. In *Erwinia carotovora, Nep* was upregulated only when the bacteria were grown on solid medium.^52^ In necrotrophic fungal pathogen *Botrytis cinerea, BcNep1* and *BcNep2* exhibit a significant upregulation at early and late stage of infection, respectively.^53^ In *P. sojae*, the expression of most of *NLP* genes was generally higher at late infection phases.^21^ In *P. capsici*, transcript levels of 6 of 11 expressed *PcNLP* genes gradually accumulated to a maximum at the late stage of infection, while the induction of remainder five *PcNLP* genes occurred at the early stages of infection.^54^ In contrast, for all *NLP* genes with detectable expression in the obligate biotroph oomycete *H. arabidopsis*, relative high expression was detected at the early stages of infection.^19^ We performed transcriptional profiling of seven *PvNLP* genes by RT-qPCR and found that all genes were expressed during very early stages of the infection. Therefore, it seems that most *NLP* genes are naturally involved in the interaction between the pathogens and their hosts, but the different expression patterns of *NLP* genes suggest their diversified roles during infection.

In this study, 6 of 7 identified PvNLP proteins are noncytotoxic which could not induce cell death in *N. benthamiana*. This is not surprising, since *P. viticola* is an obligate biotrophic pathogen that needs living host cells for completion of its life cycle. According to the expression patterns of *PvNLP* genes, it is tempting to speculate that these six nontoxic *NLP* genes play functional roles different from necrosis-inducing during the infection process and may function in development stages or primary contact between pathogen and host. It is intriguing that PvNLP7 have the ability of necrosis-inducing acting as a toxic protein though 10 out of 11 critical amino acids required for cytotoxicity are substituted in PvNLP7^18^. It may be that too many mutated amino acids have caused the protein structure to change, forming a novel conformation. And it is possible that there is a new mechanism, different from previous studies, resulting in NLP cytotoxicity which needs more in-depth research to reveal in the future. Whereas the life cycle of *P. viticola* has only a biotrophic phase, it needs living host cells for colonization. It is very likely that the phytotoxic activity of PvNLP7 expressed during physiological infection is suppressed by other effectors like RxLR protein secreted by *P. viticola*.^18,55^ Some NLPs can act as microbe-associated molecular patterns (MAMPs) which can trigger MTI.^23^ MTI is generally associated with pathogen resistance, activation of mitogen-activated protein (MAP) kinase activity, induction of ROS burst, deposition of callose, and increased expression levels of defense-related genes.^56^ In this study, we observed that expression of *PvNLP4, PvNLP5, PvNLP7*, and *PvNLP10* in *A. thaliana* enhanced resistance against *H. arabidopsidis*. And also, the PvNLP7 enhanced the expression level of defense-associated genes in *N. benthamiana*. It suggests that *PvNLP4, PvNLP5, PvNLP7*, and *PvNLP10* may each play a role as MAMP triggering MTI in Arabidopsis. It is worth noting that *PR1b* and *PR2b* genes were activated by PvNLP7. It is plausible that PvNLP7 could elicit SA signaling pathway to restrict pathogen growth. SA is the classical immunity hormone which plays key roles in plant defenses against pathogens and is generally crucial for immunity against biotrophs or hemibiotrophs.^50^ It will be very interesting to determine whether these four PvNLP proteins induced immunity can inhibit invasion of different pathogens to plants. NLPs often function as key virulence determinants of pathogens on plants due to the cytotoxic activity.^31,57^ Several *NLP* genes have been proved to be positive factors in virulence.^30,32,52^ However, three NLPs from *M. oryzae* functioned as cytolytic toxins to induce necrotic cell death the production of reactive oxygen species, but they were all dispensable for the infection of rice plants.^26^ Unfortunately, it is difficult to determine whether PvNLPs are crucial for the pathogenicity of *P. viticola* in grapevine infection because the pathogen can hardly be genetically modified due to its typical obligate lifestyle. In the further investigation, we should grope the genetic transformation system of *P. viticola*, so as to better dig out the pathogenic virulence factors of the special pathogen.

In summary, 7 *NLP* genes from *P. viticola* were characterized in detail. *PvNLP4, PvNLP5, PvNLP7*, and *PvNLP10* could enhance plants resistance to pathogens, indicating they may act as MAMPs like HaNLP3. Specifically, PvNLP7 has the dual function of stimulating defense response and cytotoxic activity. In contrast, PvNLP2, PvNLP3, and PvNLP9 are noncytotoxic proteins and have no effect on plant disease resistance. Collectively, these data demonstrated that the NLPs of *P. viticola* show a functional diversification. Moreover, these immunity-inducing NLPs will be very useful to identify novel defense-related genes against *P. viticola* or other pathogens and to lay a foundation for further study on host-pathogen interactions.

## Supplementary Material

Supplemental Material
