# Supplementary material for: Functional analysis of the Nep1-like proteins from Plasmopara viticola
Source: Plant Signal Behav. 2022 Feb 12;17(1):2000791. doi: 10.1080/15592324.2021.2000791 (PMC9176246; doi:10.1080/15592324.2021.2000791)
Supplement: Supplemental Material [file KPSB_A_2000791_SM6909.docx]

**Table S1. Primers used in this study**

| **Primer name** | **Primer sequence** | **Purpose or vector** |
| --- | --- | --- |
| ***PvNLP2-F***  ***PvNLP2-R*** | ATGTGCAAGGGATCCGGATGGGGTT | pLB vector |
|  | CTAAAGAGGGAACGCTTTTTCCAGT |  |
| ***PvNLP3-F***  ***PvNLP3-R*** | ATGGGCCCGTGGGAAGCAAAGTGGA  CTAGAACGGAAATGCTTTTTCCAGT | pLB vector |
| ***PvNLP4-F***  ***PvNLP4-R*** | ATGCAATTCGCAGTCTTTTGCCTAC  TTAAAAAGGGTACGATTCGTGGAGG | pLB vector |
| ***PvNLP5-F***  ***PvNLP5-R*** | ATGCGCGTCCTAGTATTGTGTGCCG  TTAGAAGGGTCGCGCTTTCTCAAGG | pLB vector |
| ***PvNLP7-F***  ***PvNLP7-R*** | ATGCACCTTTGTGCTCTTCTCATTG  TTAGAACGGATACGCCTCCTTCAGG | pLB vector |
| ***PvNLP9-F***  ***PvNLP9-R*** | ATGAAGATCTCCAATCTTCTCGGCG  TCACTGCAAATACTCTTTGGAGAAT | pLB vector |
| ***PvNLP10-F***  ***PvNLP10-R*** | ATGAAGTTGCCCACTTTTCTCATTG  TTAACGATCGAATGGATACGCTTTC | pLB vector |
| ***PvNLP2-AscIF***  ***PvNLP2-NotIR*** | TTGGCGCGCCATGTGCAAGGGATCCGGA | pGR106 |
|  | AAGGAAAAAAGCGGCCGCAAGAGGGAACGCTTTTT |  |
| ***PvNLP3-AscIF***  ***PvNLP3-NotIR*** | TTGGCGCGCCATGGGCCCGTGGGAAGCA  AAGGAAAAAAGCGGCCGCGAACGGAAATGCTTTTT | pGR106 |
| ***PvNLP4-AscIF***  ***PvNLP4-NotIR*** | TTGGCGCGCCATGCAATTCGCAGTCTTT  AAGGAAAAAAGCGGCCGCAAAAGGGTACGATTCGT | pGR106 |
| ***PvNLP5-AscIF***  ***PvNLP5-NotIR*** | TTGGCGCGCCATGCGCGTCCTAGTATTG  AAGGAAAAAAGCGGCCGCGAAGGGTCGCGCTTTCT | pGR106 |
| ***PvNLP7-AscIF***  ***PvNLP7-NotIR*** | TTGGCGCGCCATGCACCTTTGTGCTCTT  AAGGAAAAAAGCGGCCGCGAACGGATACGCCTCCTT | pGR106 |
| ***PvNLP9-AscIF***  ***PvNLP9-NotIR***  ***PvNLP10-AscIF***  ***PvNLP10-NotIR***  ***PvNLP2-BclIF***  ***PvNLP2-SpeIR***  ***PvNLP3-BclIF***  ***PvNLP3-SpeIR***  ***PvNLP4-BgIIIF***  ***PvNLP4-SpeIR***  ***PvNLP5-BgIIIF***  ***PvNLP5-SpeIR***  ***PvNLP7-BgIIIF***  ***PvNLP7-SpeIR***  ***PvNLP9-BclIF***  ***PvNLP9-SpeIR***  ***PvNLP10-BgIIIF***  ***PvNLP10-SpeIR***  ***PvNLP2-F(qRT)***  ***PvNLP2-R(qRT)***  ***PvNLP3-F(qRT)***  ***PvNLP3-R(qRT)***  ***PvNLP4-F(qRT)***  ***PvNLP4-R(qRT)***  ***PvNLP5-F(qRT)***  ***PvNLP5-R(qRT)***  ***PvNLP7-F(qRT)***  ***PvNLP7-R(qRT)***  ***PvNLP9-F(qRT)***  ***PvNLP9-R(qRT)***  ***PvNLP10-F(qRT)***  ***PvNLP10-R(qRT)***  ***PvActin-F***  ***PvActin-R***  ***Nb-EF1α-F***  ***Nb-EF1α-R***  ***Nb-PR1b-F***  ***Nb-PR1b-R***  ***Nb-PR2b-F***  ***Nb-PR2b-R*** | TTGGCGCGCCATGAAGATCTCCAATCTT  AAGGAAAAAAGCGGCCGCCTGCAAATACTCTTTGGA  TTGGCGCGCCATGAAGTTGCCCACTTTT  AAGGAAAAAAGCGGCCGCACGATCGAATGGATACGC  **GA**TGATCAATGTGCAAGGGATCCGGA  GGACTAGTAAGAGGGAACGCTTT  **GA**TGATCAATGGGCCCGTGGGAAGCA  GGACTAGTGAACGGAAATGCTTTT  **GA**AGATCTATGCAATTCGCAGTCTTT  GGACTAGTAAAAGGGTACGATTC  **GA**AGATCTATGCGCGTCCTAGTATTG  GGACTAGTGAAGGGTCGCGCTTT  **GA**AGATCTATGCACCTTTGTGCTCTT  GGACTAGTGAACGGATACGCCT  **GA**TGATCAATGAAGATCTCCAATCTT  GGACTAGTCTGCAAATACTCTTT  **GA**AGATCTATGAAGTTGCCCACTTTT  GGACTAGTACGATCGAATGGATA  AAGATCAATTACGAGAGT  GAACGATATTCAAAGCAT  CGTCATAGCGATGTTAGA  CGGGTTTAAGCATTACTG  TCGTGTCTCACCACTATT  GTTCTCCGTCATGTTGTC  TCGTGCGTTCTATAATTC  GTTCCTATGACCAATGTAG  CGTCACTACTGGTTATATGC  AATGTCACACCGAGAAGA  GGAGTATGCTATTGTATGGA  TGCCTTCTGAGTTAGAGA  AATGCCTGGTGTTGCTAA  AATATGATTCTGATACGACTTGAC  CGATCTCGTATCTGAATA  CTACATCATCTCATCCAT  AGAGGCCCTCAGACAAAC  TAGGTCCAAAGGTCACAA  GTGGACACTATACTCAGGTG  TCCAACTTGGAATCAAAGGG  AGGTGTTTGCTATGGAATGC  TCTGTACCCACCATCTTGC | pGR106  pGR106  pHB-flag  pHB-flag  pHB-flag  pHB-flag  pHB-flag  pHB-flag  pHB-flag  qRT-PCR  qRT-PCR  qRT-PCR  qRT-PCR  qRT-PCR  qRT-PCR  qRT-PCR  qRT-PCR  qRT-PCR  qRT-PCR  qRT-PCR |

**Table S2. Summary of the P. viticola NLP proteins analyzed in the study**

| Protein | Protein length | SignalP score | | SignalP length | Peptide sequence |
| --- | --- | --- | --- | --- | --- |
| PvNLP2 | 223 | na | na | | AIMYSWYFPKDMPSTDFGHRHDWE |
| PvNLP3 | 223 | na | na | | AIMYVWYFPKDMPSAHFGHRHDWE |
| PvNLP4 | 241 | 0.9986 | 17 | | AIMYAWYFPRDYMVTPVWVGHRNAWE |
| PvNLP5 | 239 | 0.9994 | 19 | | EIMYAWYFPRDRMITPVYIGHRNAWE |
| PvNLP7 | 241 | 0.9952 | 19 | | AIMYAWYFPKASNHIGRGVSGSRHYWL |
| PvNLP9 | 279 | 0.9850 | 19 | | AIMYAWYFPDIPLDWE |
| PvNLP10 | 273 | 0.9775 | 21 | | AIMYAWYLPRALDRVTWLVNGHRHYWL |

**Table S3. Genbank accession numbers of genes in *N. benthamiana* and *PvNLP* genes used in this study**

| Gene | Accession number |
| --- | --- |
| *PvNLP2* | MN938411.1 |
| *PvNLP3* | MN720268 |
| *PvNLP4* | MT722075.1 |
| *PvNLP5* | MT722076.1 |
| *PvNLP7* | MT722078.1 |
| *PvNLP9* | MW812346 |
| *PvNLP10* | MW812347 |
